# Cardiopulmonary arrest in Primary Care: nursing team’s theoretical, practical and soft skills interferences

**DOI:** 10.1590/0034-7167-2025-0059

**Published:** 2025-12-08

**Authors:** Caroliny Suhet Xavier Ferreira, Gabriele de Araújo Anacleto, Regina Cavalcante Agonigi, Vanessa de Almeida Ferreira Corrêa, Andressa Teoli Nunciaroni

**Affiliations:** I Universidade Federal do Estado do Rio de Janeiro. Rio de Janeiro, Rio de Janeiro, Brazil; II Secretaria Municipal de Saúde do Rio de Janeiro.Rio de Janeiro, Rio de Janeiro, Brasil

**Keywords:** Primary Health Care, Heart Arrest, Nursing Care, Cardiopulmonary Resuscitation, Nursing., Atención Primaria de Salud, Paro Cardíaco, Atención de Enfermería, Reanimación Cardiopulmonar, Enfermería.

## Abstract

**Objectives:**

to identify the determining factors and soft skills that interfere with the nursing team’s care for people in cardiorespiratory arrest in the Family Health Strategy.

**Methods:**

exploratory and descriptive research, with a qualitative approach. Thirty-five nursing professionals from two Primary Health Care units in the Brazilian capital were interviewed between March and June 2023. A categorical thematic content analysis was carried out.

**Results:**

three categories emerged: 1. Knowledge and experience as determining factors in assisting people in cardiorespiratory arrest; 2. Macro and micro work processes in Primary Health Care: from users’ arrival to advanced life support; 3. Soft skills.

**Final Considerations:**

theoretical knowledge, experience and work processes in Primary Health Care are decisive. Soft skills include leadership and communication. Continuing education strategies adapted to Primary Health Care that value soft skills are recommended.

## RESUMEN

**Objetivos:**

identificar los factores determinantes y las *soft skills* que interfieren en la atención del equipo de enfermería a las personas en paro cardiorrespiratorio en la Estrategia de Salud Familiar.

**Métodos:**

investigación exploratoria y descriptiva, con enfoque cualitativo. Se entrevistó a treinta y cinco profesionales de enfermería de dos unidades de Atención Primaria de Salud de una capital brasileña entre marzo y junio de 2023. Se realizó un análisis de contenido temático categórico.

**Resultados:**

emergieron tres categorías: 1. Conocimiento y experiencia como factores determinantes en la atención a las personas en parada cardiorrespiratoria; 2. Macro y micro procesos de trabajo en Atención Primaria de Salud: desde la llegada del usuario hasta el soporte vital avanzado; 3. Habilidades comportamentales: *soft skills*.

**Consideraciones Finales:**

los conocimientos teóricos, la experiencia y el proceso de trabajo en Atención Primaria son decisivos. Las *soft skills* incluyen liderazgo y comunicación. Se recomiendan estrategias de formación continua adaptadas a la Atención Primaria que valoren las *soft skills*.

## INTRODUCTION

Caring for a person in cardiorespiratory arrest (CPA) is an emergency situation that requires a quick and efficient response. There is a consensus and a high degree of evidence that the faster chest compression maneuvers are started, the better the patients’ prognosis^(1)^.

The incidence of cardiac arrest in the out-of-hospital setting in the United States and Europe ranges from 50 to 100 cases per 100,000 population per year. The variation depends on the emergency system quality and on demographic and public health differences^(2)^. In Brazil, this incidence is a critical statistic, since the recording of exact data varies according to different regions. It is known, however, that the main rhythm of out-of-hospital CPA is ventricular fibrillation, corresponding to approximately 80% of cases. The survival rate for shockable rhythms is 50% to 70%, when shock is administered between three and five minutes after the onset of CPA, while for non-shockable rhythms, this rate is less than 17%^(3)^.

In an out-of-hospital setting, different studies suggest that community-based initiatives contribute to the faster implementation of care for people in cardiac arrest, in addition to improving survival and post-arrest clinical outcomes^(4)^. These are community interventions focused on improving cardiopulmonary resuscitation skills among bystanders of a cardiac arrest, the Take Heart America and TAKE10 programs^(5)^, Project HeartRescue^(6)^, the Lifesavers campaign^(7)^ and the World Restart a Heart initiative^(8)^.

In Brazil, such initiatives are still progressing slowly, both due to cultural diversity and health literacy and the complex and multiple organization of the health network at a national level^(9)^. However, Primary Health Care (PHC) plays a vital role in the implementation and assessment of actions with community approaches that bring together lay people and healthcare professionals, which can be translated into the initial response to an out-of-hospital CPA.

Since they are present in all Brazilian regions, located close to homes and strengthen the bond between community and professionals, PHC’s professional skills can be extrapolated to the community context. Moreover, PHC is the main access to the Health Care Network and is also responsible for coordinating care for people in cardiac arrest situations, as it is part of the Emergency Network.

The health actions developed in PHC have unique objectives that go beyond addressing a specific complaint or problem, as they consider subjective, family, community and social aspects, with emergency care not being their main focus. Caring for people in cardiac arrest in PHC requires preparation, adequate training, availability of essential resources and effective soft skills^(10)^.

The recognition of the specificities of this point of the Health Care Network, associated with infrastructure, flows, training needs and interpersonal skills, can contribute to promoting effective actions of continuing education, organization and access to the necessary materials and clinical protocols that support the team’s work in basic and advanced life support^(11,12)^.

To enhance PHC role as a first responder to people in cardiac arrest and strengthen community-based actions in out-of-hospital emergency care, it is necessary to know the determining factors and necessary individual and interpersonal soft skills. Such skills refer to non-technical and non-professionally specific competencies that allow a person to relate effectively with others and perform their work effectively^(13)^.

Soft skills are generally related to an individual’s behavior, attitude, communication, empathy, and emotional intelligence, associated with interpersonal relationship skills. These skills are also necessary for effective care for people in cardiac arrest, especially in primary care, where there is a family and community approach, and this type of emergency may not be frequent. Recognizing soft skills can be crucial to supporting the implementation of actions to continue post-cardiac arrest care and recovery.

A scoping review that included 12 studies identified, despite the scarcity of literature, that leadership, teamwork and communication are the priority soft skills for professional care for people in out-of-hospital cardiac arrest situations^(14)^. However, this review does not include Brazilian studies or PHC units that make up the Health Care Network, such as the Brazilian Health System. This gap points to the need to understand these skills and their determining factors in PHC context.

Thus, the present study is pioneering, and, based on the experiential approach proposed by Sidani and Braden^(15)^, it aims to contribute to strengthening PHC nursing as a “preceptor” in community training to achieve objectives related to rapid care for people in CPA situations and better clinical outcomes.

## OBJECTIVES

To identify the determining factors and soft skills that interfere with the nursing team’s care for people in a CPA situation in the Family Health Strategy (FHS).

## METHODS

### Ethical aspects

The research was approved by the Research Ethics Committee of the proposing institution and the Municipal Health Department where the research was conducted. The municipality name was kept confidential for the ethical preservation of participants and the units to which they are linked. The Informed Consent Form was obtained from all individuals involved in the study through a written form. Participants were identified by the letter E, followed by an Arabic numeral.

### Study design

This is descriptive exploratory research with a qualitative approach. The Standards for Reporting Qualitative Research were used to write the study^(16)^.

### Scenario and participants

The research was conducted with 35 nursing professionals (nurses and nursing technicians) from two health units with FHS teams, located in a large Brazilian city (over 1.5 million inhabitants). The health units were chosen through non-probabilistic sampling, due to the technical cooperation already existing between the research team and healthcare professionals from the units.

Both units are close to emergency units (within a radius of up to 5 km), but occasionally serve people who are spontaneously needed in urgent and emergency situations. The researchers have clinical and teaching experience in the context of PHC as well as in caring for people in cardiac arrest situations and training in cardiopulmonary resuscitation (CPR).

Nurses or nursing technicians from the FHS who work in one of the health units defined as the study setting were included. Professionals who were away from their professional activities during the data collection period were excluded.

No sample calculation was performed, as this is a finite sample, since data collection fields were the limiting factors. To reduce bias in participant selection, two different health units were considered and all professionals (n=59) who met the inclusion criteria were invited. The researchers invited participants personally.

### Data source, collection and organization

Data collection took place between March and June 2023, through semi-structured individual interviews, in which an instrument was used to record participant personal and professional characterization and a script with six triggering questions, prepared by the authors, related to the object of study, divided into two major domains: the experience in providing care for CPA in PHC unit; and the critical perception of this experience with an emphasis on aspects related to human talent and interpersonal skills.

Data collection was completed when all professionals available at the unit during the period defined for data collection were interviewed. The theoretical data saturation technique was not used to conclude the research, even though some responses presented a repetitive pattern, since the study intended to value participant’s individual and unique experiences.

Data were collected individually after signing the Informed Consent Form at a location chosen by participants. The interviews were audio-recorded, lasting approximately ten minutes, and were later transcribed into the research team’s personal database.

### Data analysis

Bardin’s methodological framework was used with the systematization proposed by Oliveira^(17)^: pre-analysis; material exploration; and treatment of results. After the interviews were transcribed, a quick reading of analysis *corpus* was performed to begin the selection of registration units (RUs), identified by means of phrases and thematic units. The material exploration stage involved the coding of 523 RUs, aggregating the construction of 21 units of meaning (UMs) and the recording of these units in tables (supplementary material). Then, the UMs were grouped into three thematic categories.

## RESULTS

The sample consisted of 26 nurses and nine nursing technicians. Table 1 presents participant characteristics.

**Table 1:** Participant characterization in two Primary Health Care units, Brazil, 2024 (N=35)

Variable	Total n (%)
Female	32 (91.4%)
**Age range** 18 to 30 years 31 to 40 years 41 to 50 years 51 to 60 years Over 60 years	9 (25.7%) 13 (37.1%) 7 (20%) 5 (14.3%) 1 (2.9%)
**Professional category** Nurse Nursing technician	26 (74.3%) 9 (25.7%)
**Training time** Less than 1 year 1 to 5 years 6 to 10 years 11 to 15 years 16 to 20 years More than 20 years	1 (2.9%) 15 (42.9%) 5 (14.3%) 6 (17.1%) 5 (14.3%) 3 (8.6%)
**Time working in PHC** Less than 1 year 1 to 5 years 6 to 10 years 11 to 15 years 16 to 20 years More than 20 years	7 (20%) 15 (37.1%) 6 (17.1%) 3 (14.3%) 2 (5.7%) 2 (5.7%)
**Previously worked in hospital care**	20 (57.1%)
**Witnessed a CPA**	26 (74.3%)
**Participated in a CPR**	19 (54.3%)
**Completed prior training in BLS**	33 (94.3%)
**Completed prior training in ALS**	8 (22.9%)

*PHC - Primary Health Care; CPA - cardiorespiratory arrest; CPR - cardiopulmonary resuscitation; BLS - basic life support; ALS - advanced life support.*

From the analysis of categorical thematic content, four thematic categories were obtained, which will be presented below:

### Knowledge and experience as determining factors in caring for people in cardiorespiratory arrest

This category is composed of 182 RUs (34.8% of the total RUs in the analysis *corpus*) and divided into two subcategories: “Technical knowledge and previous experience as determining factors for the success of cardiopulmonary resuscitation”; and “Continuing education as a professional qualification strategy”. It encompasses four RUs: “technical knowledge as a booster of CPR care” (64 RUs); “previous experience in CPR and hospital experience as determining factors for care” (14 RUs); “non-occurrence of CPR and other emergencies on a routine basis in PHC” (33 RUs); and “need for periodic training of PHC professional team” (71 RUs).

The following RUs highlight participants’ statements regarding the thematic category described:

*If you are not aware of that action, of the event, you cannot have any differential behavior.* (E2)

*First, you need to know and have mastery of the knowledge, and not just the professional, but all the teams involved, to master the subject. I think this is essential for excellent service.* (E8)

Professionals showed that having previous hospital experience or CPR experience is a determining factor for the success of CPA care in PHC, even though the period of experience was reported as between one and five years for the majority of participants (57.9%).

*It’s a characteristic here. We have few professionals who don’t have hospital experience, who don’t work in hospitals. But professionals who graduated from college went into primary care, never had hospital experience, never experienced this, so it won’t work, the work there will be completely dysfunctional, it will be completely lost.* (E10)

Regarding the challenges of providing this service in PHC, participants presented periodic training as a determining factor:

*Lack of periodic training, so in the municipality of* [omitted for ethical reasons], *there is a lot of turnover in PHC, so it would be interesting for this training to happen regularly, not so spaced out as it is. I think this could be improved and not focus only on healthcare professionals, but on the professionals in the unit, from the access controller to people who work in cleaning and administration as well.* (E23)

*Daily training, which we often can’t do because it’s too demanding.* (E1)

Although most participants stated that they had already participated in some CPR (54.3%) and had received prior training in basic life support (94.3%), their statements reveal their insecurity in providing this type of care in the context of PHC. One of the factors that participants attribute to this result is related to the perception that this is not a routine situation at this point of care.

*Mainly because it is a basic care unit, so we don’t have to deal with this type of situation, let’s say.* (E5)

*Because it is not something that happens routinely. So, since it does not happen frequently in our practice, sometimes, if it happens suddenly, there may be some failure due to it.* (E11)

*Since we don’t experience this often, it can sometimes be a bit complicated to have that well-qualified professional who knows exactly what to do.* (E33)

*Have continuous training, because, as it is not a routine here, it is not something that happens all the time, so professionals end up not having the agility for the situation.* (E9)

### Macro and micro work processes in Primary Health Care: from users’ arrival to advanced life support

This category presents the work process as a determining factor in the care of people in cardiac arrest. Composed of 159 RUs (30.4% of the total RUs), it was constructed from subcategories entitled: “Macroprocesses: service routine, work demand and material and structural resources”; and “Microprocesses: work environment organization for the care of people in cardiorespiratory arrest”. Its composition includes six RUs, described below.

The “availability of material and adequate physical structure at the BHU” (84 RUs) UM encompasses participants’ statements regarding the need for materials and infrastructure so that the service is provided quickly and efficiently:

*First thing, organization of materials and medicines, and that the entire team is aware of where the materials are.* (E11)

*Knowing where the materials are, the structure of the unit, having materials available, easy location, easy access, visualization, those things.* (E14)

The “service routine and work process in PHC” (13 RUs) and “work demand in PHC routine” (seven RUs) UMs highlight the service routine as a determining factor for care, in addition to showing that work demand also interferes in this process:

*Here, for instance, there is a very high turnover of professionals, so we don’t know how prepared they are.* (E18)

*We are in the office doing prenatal care, childcare, preventive care, and then we send a patient who is feeling unwell, and then we have to rush down to determine whether it is a seizure or not. It is difficult for the staff who receive care down there.* (E22)

The “sector organization and flow of CPA care” (32 RUs) UM addresses the micro work process inherent to the care itself, whose determining factors can speed up or delay care:

*If you don’t stop and think, organize your thoughts to be able to complete all these steps, you won’t get anywhere, you’ll get stuck, and then you’ll disrupt the entire flow. Exactly, all the other steps.* (E13)

*So, like this, the parade, for instance, has to have a dynamic where everyone knows their role.* (E5)

The “lack of knowledge and disorganization of material and its storage as a challenge in service” (13 RUs) UM refers directly to the necessary materials and equipment:

*Check that all the materials are complete and separated in advance. The professional in charge must always be making a checklist to avoid any complications during the service.* (E8)

The “early identification of the risk framework” (ten RUs) UM demonstrates that the organization of the work microprocess begins with the early identification of CPA:

*I think what scares me the most when it comes to primary care is the delay that can occur when the patient arrives at the front feeling unwell and comes to* […] [the sentence is not completed by the participant]. *We spend a lot of time in the office, right?* (E10)

### Soft skills

This category consists of 182 RUs (34.8% of total RUs) and is divided into two subcategories. Subcategory 1, “Interfaces between leadership and communication in cardiopulmonary arrest care”, consists of six RUs: “welcoming and qualified listening” (three RUs); “teamwork and good interpersonal relationships” (23 RUs); “importance of effective communication among the team for successful CPR” (ten RUs); “assignment of CPR leadership to a professional category, mainly nurses and physicians, or to those who initiate care” (28 RUs); “experience and preparation as essential factors for leadership” (18 RUs); and “leadership as an essential skill during care” (19 RUs).

The following RUs highlight the participants’ statements regarding the subcategory described:

*Sometimes you are in a basic unit where you sometimes notice a difficulty in relationships between teams, between professionals and I think that at this moment, regardless of adversities, this cannot happen. It is about forgetting any adversity and focusing on what is happening there.* (E8)

*And I think that mainly communication between the multidisciplinary team is needed to act in this type of scenario.* (E17)

*So, I think that the nurse has to be* [the leader], *because the nurse is already used to this, is already used to managing everything. So, we already have this practice, it’s ours, because the nurse knows the procedure room, the nurse knows where all the materials are, the nurse knows all the technicians who are here, knows the technician who is good at getting a vein, knows the technician who is good at doing anything, knows everyone’s name, “so-and-so does this, so-and-so does this”. Not everyone, not all categories will know how to do this.* (E10)

*The leader? Professional competence, he becomes a leader. He is prepared, as I said. He has to have complete knowledge of what he is doing to guide us, the technicians, to do it correctly.* (E31)

Subcategory 2 “Soft skills in cardiopulmonary resuscitation in Primary Health Care” is composed of five RUs: “the importance of agility in the success of CPR” (22 RUs); “having confidence to perform care” (ten RUs); “having emotional control during care” (28 RUs); “the lack of belonging of PHC professionals in the RUE and in the links of the survival chain” (17 RUs); and “need for focus and attention in CPR care” (four RUs).

The following assertions corroborate the subcategory described:

*I think proactivity. Agility so you can think quickly, so you can act quickly. Attention, focus. I think that’s it.* (E22)

*Calm. Calm and knowing how to reason, because in times of desperation, people get all worked up and it doesn’t work out. Unfortunately, in a super chaotic moment, you’re going to have to be the calm person; you have to have two calm people. Calm in the sense of knowing how to direct. Of course, there’s tension, but giving the command and knowing how to deal with it. So, I don’t think that’s even calm, let’s say, it’s really knowing how to direct. Security has to convey security to everyone who’s there.* (E20)

*Because normally, we guide the population, but the population doesn’t know that this is not an emergency or urgent care place. So, if they stop, they come here. It’s difficult.* (E5)

Chart 1 (supplementary material) summarizes the elaboration of the four thematic categories, including the RUs and UMs, obtained from content analysis.

### DISCUSSION

This study presents the determining factors and soft skills in the care of people in a CPA situation in PHC. This is a context little explored for this type of theme and practice, but with great potential to reverse the CPA situation, since it is located close to the residence and organized in a community manner.

Professionals’ insecurity can be justified because it is known that the less frequent the contact, the lower the retention of knowledge and skills^(18)^. Sporadic training may not be enough for effective action in CPA care^(19,20)^. Therefore, theoretical-practical continuing education actions are recommended with greater frequency and less intensity of new knowledge - “low dose - high frequency model”^(21)^.

In addition to the theoretical approach and practice of the CPR technique, our results indicate that continuing education strategies for healthcare professionals need to include plans that enable the acquisition of soft skills applied to the workplace and available resources, without imposing excessive pressure. Possibilities for innovation include the inclusion of patients and families to empower professionals^(22)^, the integration of face-to-face meetings and the use of technologies such as videos and remote classes^(23)^, the use of interactive games^(24)^, and smart devices, such as cell phones/apps or watches^(25)^.

The “Macro and micro work processes in Primary Health Care: from users’ arrival to advanced life support” category highlights the availability of materials, adequate infrastructure, service routine and work process in PHC as the main determining factors. The work demand in the unit’s routine, the sector organization and the flow of care for CPA, early identification of the risk situation, and lack of knowledge about the material and its storage as challenges in care stand out.

There is consensus that the availability of materials and equipment is an indispensable factor in assisting victims of cardiac arrest. Therefore, it is essential to ensure the necessary supply and organization. However, insufficient materials or supplies should not prolong or interrupt resuscitation^(20)^. The actions performed during the initial minutes of CPA care are critical to survival; therefore, basic life support must be performed appropriately and quickly^(1)^. There is, therefore, a co-responsibility between institutional action and individual and interpersonal soft skills.

The last category grouped soft skills, which are soft skills - individual and social - related to the way professionals deal with others and with themselves in different situations. They are often grouped into three categories: social skills, such as leadership; cognitive skills, such as situational awareness; and personal resource factors, such as decision-making in stressful situations^(26)^.

In the context of CPA care in PHC, these skills can determine the efficiency of care, which changes clinical outcomes and professional satisfaction^(27)^. In this study, soft skills related to leadership, agility, security, emotional control, focus, attention and a sense of belonging emerged.

Participants pointed out leadership as an essential factor for successful care. Professionals with technical knowledge are more confident in leading the process in a qualified manner. The emergency care’s success team depends on the efficiency of a leader to guide the professionals involved in care^(28)^.

There is a direct relationship between the leader’s communication skills and the team’s performance, where effective communication, technical skills and good interpersonal relationships make up the triad necessary for effective leadership in conducting CPR^(28)^. As evidenced in the interviews, the literature also points to nurses as a fundamental link in team integration and organization, facilitating work processes, in addition to participating in the activities developed and providing theoretical support to members through continuing education^(29)^.

Agility is required in clinical protocols for CPA care, both for rapid recognition of the situation and for initiation of CPR and early defibrillation when indicated. Agility is closely related to the safety of performing an action and the focus on what is being implemented. These skills become even more important in the context of PHC, since the health unit is not always prepared to handle emergencies.

Advancing training with a view to promoting the acquisition of soft skills is a contemporary challenge that has been the subject of recent studies^(30-32)^. In this regard, constant professional updating and the analysis of the incorporation of different methods for the development of non-technical skills in CPA care are potential fields for future research.

Furthermore, including activities that enable the development of soft skills in care for people in CPR situations in PHC deserves attention in educational settings and public health policies. Furthermore, the facilitator of training aimed at continuing education for CPR must also rely on soft skills^(26)^.

### Study limitations

The study has limitations such as the inclusion of only two FHS units, which are located relatively close to emergency care units, which supposedly leads to a reduction in the experience of CPA care by participants. Furthermore, a methodological limitation refers to the lack of confirmation of the data collected after transcription with participants.

### Contributions to nursing, health or public policy

This research highlights the fragility of the development of soft skills among PHC nursing professionals for the care of people in cardiac arrest situations. In this regard, it is a current challenge to improve public policies and training centers with appropriate and innovative methodologies that enable the acquisition of non-technical skills for basic and advanced life support. Active teaching methodologies, especially realistic health simulation, can make great contributions to overcoming this challenge.

The results of this research may support the development of interventions aimed at improving PHC professionals’ theoretical-practical knowledge and soft skills, directing the continuing education actions of teams and the implementation of actions by stakeholders linked to the Health Care Network. Furthermore, this study points out the skills necessary for caring for people in CPA in the context of PHC, based on identification by the nursing staff themselves, in order to guide future studies that assess the different methodologies and outcomes related to CPR.

## FINAL CONSIDERATIONS

This study identified thematic categories related to the determining factors and soft skills in the perception of the nursing team working in PHC in the face of CPR situations. The importance of leadership, effective communication, fast, agile and focused decision-making, and work process organization are highlighted as fundamental non-technical soft skills for the success of CPR in this context. The co-responsibility for the success of care for people in CPR situations in PHC is also evidenced by the need for the availability of materials, adequate infrastructure, service routine and work process organization. Studies that assess soft skills (behavioral and interpersonal skills) in different social contexts are encouraged.

## Data Availability

The research data are available only upon request.

## References

[1] Perman SM, Elmer J, Maciel CB, Uzendu A, May T, Mumma BE (2023). 2023 American Heart Association Focused Update on Adult Advanced Cardiovascular Life Support: An Update to the American Heart Association Guidelines for Cardiopulmonary Resuscitation and Emergency Cardiovascular Care. Circulation.

[2] Virani SS, Alonso A, Benjamin EJ, Bittencourt MS, Callaway CW, Carson AP (2020). Heart Disease and Stroke statistics-2020 Update. Circulation.

[3] Salim TR, Soares GP. (2023). Outcome Analysis after Out-of-Hospital Cardiac Arrest. Arqu Bras Cardiol.

[4] Yu Y, Meng Q, Munot S, Nguyen TN, Redfern J, Chow CK. (2020). Assessment of Community Interventions for Bystander Cardiopulmonary Resuscitation in Out-of-Hospital Cardiac Arrest. JAMA Network Open.

[5] Lick CJ, Aufderheide TP, Niskanen RA, Steinkamp JE, Davis SP, Nygaard SD (2011). Take Heart America: a comprehensive, community-wide, systems-based approach to the treatment of cardiac arrest. Crit Care Med.

[6] van Diepen S, Abella BS, Bobrow BJ, Nichol G, Jollis JG, Mellor J (2013). Multistate implementation of guideline-based cardiac resuscitation systems of care: description of the Heart. Rescue Project.

[7] Perkins GD, Lockey AS, Belder MA, Moore F, Weissberg P, Gray H. (2015). National initiatives to improve outcomes from out-of-hospital cardiac arrest in England. Emergency Med J.

[8] Böttiger BW, Lockey A, Aickin R, Castren M, Caen A, Escalante R (2018). “All citizens of the world can save a life” : the World Restart a Heart (WRAH) initiative starts in 2018. Resuscitation.

[9] Bartlett ES, Flor LS, Medeiros DS, Colombara DV, Johanns CK, Camargo Vaz FA (2020). Public knowledge of cardiovascular disease and response to acute cardiac events in three municipalities in Brazil. Open Heart.

[10] Smith A, Masters S, Ball S, Finn J. (2023). The incidence and outcomes of out-of-hospital cardiac arrest in metropolitan versus rural locations: a systematic review and meta-analysis. Resuscitation.

[11] Castôr KS, Nonato P, Sabino LS, Ximenes G, Merubia N. (2023). Atendimento a pessoas com parada cardiorrespiratória na ESF. Braz J Implantol Health Sci.

[12] Bitencourt AC, Rennó GM. (2023). Suporte básico de vida na atenção primária à saúde: revisão integrativa da literatura. Rev Enferm Atenção Saúde.

[13] Siu JLR, Salazar RER, Montaño LF. (2021). Habilidades blandas y el desempeño docente en el nivel superior de la educación. Propósitos Represent.

[14] Cormack S, Scott S, Stedmon A. (2020). Non-technical skills in out-of-hospital cardiac arrest management: a scoping review. Austral J Paramed.

[15] Sidani S, Braden CJ. (2021). Intervenções de enfermagem e saúde: design, avaliação e implementação.

[16] O’Brien BC, Harris IB, Beckman TJ, Reed DA, Cook DA. (2014). Standards for reporting qualitative research. Acad Med.

[17] Oliveira DC. (2008). Análise de conteúdo temático-categorial: uma proposta de sistematização. Rev Enferm UERJ.

[18] Bukiran A, Erdur B, Ozen M, Bozkurt AI. (2014). Retention of nurses’ knowledge after basic life support and advanced cardiac life support training at immediate, 6-month, and 12-month post-training intervals: a longitudinal study of nurses in Turkey. J Emergency Nurs.

[19] Greif R, Lockey AS, Conaghan P, Lippert A, De Vries W, Monsieurs KG (2015). European Resuscitation Council Guidelines for Resuscitation 2015. Resuscitation.

[20] Cheng A, Nadkarni VM, Mancini MB, Hunt EA, Sinz EH, Merchant RM (2018). Resuscitation education science: educational strategies to improve outcomes from cardiac arrest: a scientific statement from the American Heart Association. Circulation.

[21] Anderson R, Sebaldt A, Lin Y, Cheng A. (2019). Optimal training frequency for acquisition and retention of high-quality CPR skills: a randomized trial. Resuscitation.

[22] Low WR, Sandercock GRH, Freeman P, Winter ME, Butt J, Maynard I. (2021). Pressure training for performance domains: a meta-analysis. Sport, Exerc Perform Psychol.

[23] Lactona ID, Suryanto S. (2021). Efficacy and knowledge of conducting CPR through online learning during the COVID-19 pandemic: a literature review. J Public Health Res.

[24] Siqueira TV, Nascimento JSG, Oliveira JLG, Regino DSG, Dalri MCB. (2020). The use of serious games as an innovative educational strategy for learning cardiopulmonary resuscitation: an integrative review. Rev Gaúcha Enferm.

[25] Huang LW, Chan YW, Tsan YT, Zhang QX, Chan WC, Yang HH. (2024). Implementation of a smart teaching and assessment system for high-quality cardiopulmonary resuscitation. Diagnostics.

[26] Sylvia LM. (2024). Are soft skills “soft”? importance of soft skills in facilitating optimal patient care. Am J Health-Sys Pharm.

[27] Gabr AK. (2019). The importance of nontechnical skills in leading cardiopulmonary resuscitation teams. J Royal Coll Phys Edinburgh.

[28] Smith SC, Kazi DS, Anderson CAM, Beam C, Boyle DS, Carter KG (2024). International Initiatives of the American Heart Association: original concepts, present programs, and future focus: a science advisory from the American Heart Association. Circulation.

[29] Armstrong P, Peckler B, Pilkinton-Ching J, McQuade D, Rogan A. (2020). Effect of simulation training on nurse leadership in a shared leadership model for cardiopulmonary resuscitation in the emergency department. Emergency Med Austral.

[30] O’Keeffe DA, Losty M, Traynor O, Doherty EM. (2020). Objective assessment of surgical trainees’ non-technical skills: improved performance following a two-year program of instruction. Am J Surg.

[31] Pradarelli JC, Gupta A, Lipsitz S, Blair PG, Sachdeva AK, Smink DS (2020). Assessment of the Non-Technical Skills for Surgeons (NOTSS) framework in the USA. Brit J Surg.

[32] Gaska K, Pavlinec C, Cebula G, Pisarska-Adamczyk M, Szopa M. (2023). Non-technical Skills in Cardiopulmonary Resuscitation: improvement and evaluation of a new course introduced to the curriculum at a Medical School in Poland in 2018 to 2019. SAGE Open.

