# Supplementary material for: Cardiopulmonary arrest in Primary Care: nursing team’s theoretical, practical and soft skills interferences
Source: Rev Bras Enferm. 2025 Dec 8;78(5):e20250059. doi: 10.1590/0034-7167-2025-0059 (PMC12707765; doi:10.1590/0034-7167-2025-0059)
Supplement: Translation

# Translation: Parada cardiorrespiratória na Atenção Primária: interferências teóricas, práticas e *soft skills* da equipe de enfermagem

**DOI:** 10.1590/0034-7167-2025-0059pt

**Authors:** Caroliny Suhet Xavier Ferreira, Gabriele de Araújo Anacleto, Regina Cavalcante Agonigi, Vanessa de Almeida Ferreira Corrêa, Andressa Teoli Nunciaroni

**Affiliations:** I Universidade Federal do Estado do Rio de Janeiro. Rio de Janeiro, Rio de Janeiro, Brasil; II Secretaria Municipal de Saúde do Rio de Janeiro.Rio de Janeiro, Rio de Janeiro, Brasil

**Keywords:** Atenção Primária à, Saúde, Parada Cardíaca, Cuidados de Enfermagem, Reanimação Cardiopulmonar, Enfermagem.

## Abstract

**Objetivos:**

identificar os fatores determinantes e *soft skills* que interferem no atendimento da equipe de enfermagem à pessoa em situação de parada cardiorrespiratória na Estratégia Saúde da Família.

**Métodos:**

pesquisa exploratória e descritiva, de abordagem qualitativa. Foram entrevistados 35 profissionais de enfermagem de duas unidades de Atenção Primária à Saúde de capital brasileira entre março e junho de 2023. Realizou-se análise de conteúdo temático categorial.

**Resultados:**

três categorias emergiram: 1. Conhecimento e experiência como fatores determinantes da assistência à pessoa em situação de parada cardiorrespiratória; 2. Macro e micro processos de trabalho na Atenção Primária à Saúde: da chegada do usuário ao suporte avançado de vida; 3. Habilidades comportamentais: *soft skills*.

**Considerações Finais:**

o conhecimento teórico, experiência e processo de trabalho na Atenção Primária à Saúde são determinantes. *Soft skills* incluem liderança e comunicação. Estratégias de educação permanente adaptadas para Atenção Primária à Saúde que valorizem *soft skills* são recomendadas.

## INTRODUÇÃO

O atendimento à pessoa em parada cardiorrespiratória (PCR) é uma situação de emergência que requer resposta rápida e eficiente. É consenso e com elevado grau de evidência que quanto mais rápido ocorre o início das manobras de compressão torácica, melhor o prognóstico do paciente^(1)^.

A incidência da PCR no ambiente extra-hospitalar nos Estados Unidos e na Europa varia entre 50 e 100 casos por 100.000 habitantes por ano. A variação depende da qualidade do sistema de emergência e das diferenças demográficas e de saúde pública^(2)^. No Brasil, essa incidência corresponde a uma estatística crítica, pois o registro de dados exatos varia de acordo com diferentes regiões. Sabe-se, porém, que o principal ritmo de PCR extra-hospitalar é a fibrilação ventricular, correspondendo a cerca de 80% dos casos. A taxa de sobrevivência para ritmos chocáveis é de 50% a 70%, quando o choque é administrado entre três e cinco minutos do início da PCR, enquanto para ritmos não chocáveis, essa taxa é inferior a 17%^(3)^.

No contexto extra-hospitalar, diferentes estudos sugerem que iniciativas de abordagem comunitária contribuem para que o atendimento à pessoa em situação de PCR seja implementado mais rapidamente, além de melhorar a sobrevida e desfechos clínicos pós-parada^(4)^. São intervenções comunitárias, com foco em melhorar habilidades de ressuscitação cardiopulmonar entre espectadores de uma PCR, os programas *Take Heart America* e TAKE10^(5)^, o *Project HeartRescue* ^(6)^, a campanha *Lifesavers* ^(7)^ e a iniciativa *World Restart a Heart* ^(8)^.

No Brasil, tais iniciativas ainda avançam de forma lenta, tanto pela diversidade cultural e de letramento em saúde quanto pela complexa e múltipla organização da rede de saúde em nível nacional^(9)^. No entanto, a Atenção Primária à Saúde (APS) ocupa papel vital na implementação e avaliação de ações com abordagens comunitárias que aproximam pessoas leigas e profissionais de saúde, o que pode ser transcrito para a resposta inicial a uma PCR extra-hospitalar.

Por estarem presentes em todas as regiões brasileiras, localizarem-se próximas às residências e potencializarem o vínculo entre comunidade e profissionais, as habilidades profissionais da APS podem extrapolar para o contexto comunitário. Além disso, a APS configura-se o principal acesso à Rede de Atenção à Saúde e também é responsável pela coordenação do atendimento à pessoa em situação de PCR, visto que compõe a Rede de Urgências e Emergências.

As ações de saúde desenvolvidas na APS possuem objetivos singulares que ultrapassam o atendimento de uma queixa ou problema específico, pois consideram aspectos subjetivos, familiares, comunitários e sociais, não sendo o atendimento a emergências seu foco principal. O atendimento à pessoa em situação de PCR na APS exige preparação, treinamento adequado, disponibilidade de recursos essenciais e habilidades comportamentais eficazes^(10)^.

O reconhecimento das especificidades deste ponto da Rede de Atenção à Saúde, associadas a infraestrutura, fluxos, necessidades de capacitações e habilidades interpessoais, pode contribuir para a promoção de ações efetivas de educação permanente, organização e acesso aos materiais necessários e protocolos clínicos que apoiem o trabalho da equipe no suporte básico e avançado de vida^(11,12)^.

Para potencializar a atuação da APS como primeiro atendimento à pessoa em situação de PCR e fortalecer as ações de base comunitária no cuidado de emergência extra-hospitalar, faz-se necessário conhecer os fatores determinantes e habilidades comportamentais individuais e interpessoais necessárias (*soft skills*). Tais habilidades referem-se às competências não técnicas e não específicas de uma profissão que permite que uma pessoa se relacione efetivamente com os outros e execute seu trabalho de maneira eficaz^(13)^.

As habilidades compreendidas como *soft skills* são geralmente relacionadas ao comportamento, atitude, comunicação, empatia e inteligência emocional de um indivíduo, associadas às capacidades de relações interpessoais. Tratam-se de habilidades também necessárias para a efetiva atuação no atendimento à pessoa em situação de PCR, sobretudo na APS, onde existe uma abordagem familiar e comunitária, e esse tipo de emergência pode não ser frequente. Reconhecer as *soft skills* pode ser determinante para apoiar a implementação de ações de continuidade da assistência pós-PCR e recuperação.

Revisão de escopo que incluiu 12 estudos identificou, apesar de escassa literatura, que liderança, trabalho em equipe e comunicação são as *soft skills* prioritárias para o atendimento profissional à pessoa em situação de PCR extra-hospitalar^(14)^. No entanto, esta revisão não contempla estudos brasileiros ou unidades de APS que compõem a Rede de Atenção à Saúde como no Sistema Único de Saúde. Tal lacuna aponta para a necessidade de se conhecer essas habilidades e seus fatores determinante*s* no contexto na APS.

Dessa forma, o presente estudo é pioneiro, sendo que, a partir da abordagem experiencial proposta por Sidani e Braden^(15)^, visa contribuir para o fortalecimento da enfermagem da APS como “preceptora” na formação comunitária para o alcance de objetivos relacionados ao rápido atendimento à pessoa em situação de PCR e melhores desfechos clínicos.

## OBJETIVOS

Identificar os fatores determinantes e as *soft skills* que interferem no atendimento da equipe de enfermagem à pessoa em situação de PCR na Estratégia Saúde da Família (ESF).

## MÉTODOS

### Aspectos éticos

A pesquisa foi aprovada pelo Comitê de Ética em Pesquisa da instituição proponente e da Secretaria Municipal de Saúde onde a pesquisa foi realizada. O nome do município foi mantido em sigilo para preservação ética dos participantes e unidades às quais são vinculados. Foi obtido o Termo de Consentimento Livre e Esclarecido de todos os indivíduos envolvidos no estudo por meio de um termo escrito. Os participantes foram identificados pela letra E, seguida de algarismo arábico.

### Tipo de estudo

Trata-se de pesquisa exploratória descritiva, com abordagem qualitativa. Para a redação do estudo, utilizou-se o *Standards for Reporting Qualitative Research* ^(16)^.

### Cenário e participantes

A pesquisa foi realizada com 35 profissionais de enfermagem (enfermeiras e técnicas de enfermagem) de duas unidades de saúde com equipes da ESF, localizadas em um município brasileiro de grande porte (mais de 1,5 milhões de habitantes). A escolha das unidades de saúde ocorreu por meio de amostragem não probabilística, devido à cooperação técnica já existente entre equipe de pesquisa e profissionais de saúde das unidades.

Ambas as unidades possuem proximidade com unidades de emergência (raio de até 5 km), mas atendem, esporadicamente, pessoas por demanda espontânea em situações de urgência e emergência. As pesquisadoras possuem experiência clínica e de ensino no contexto da APS, além de no atendimento à pessoa em situação de PCR e treinamento em reanimação cardiopulmonar (RCP).

Foram incluídas enfermeiras ou técnicas de enfermagem da ESF que atuem em uma das unidades de saúde definidas como cenário do estudo. Foram excluídas as profissionais afastadas da atividade profissional no período de coleta de dados.

Não foi realizado cálculo amostral, pois trata-se de amostra finita, uma vez que os campos de coleta de dados foram os fatores limitantes. Para diminuir os vieses na seleção dos participantes, foram consideradas duas unidades de saúde distintas e convidados todos os profissionais (n=59) que atendessem aos critérios de inclusão. As pesquisadoras convidaram os participantes pessoalmente.

### Fonte, coleta e organização de dados

A coleta de dados ocorreu entre março e junho de 2023, por meio de entrevista individual semiestruturada, em que utilizou-se um instrumento para o registro da caracterização pessoal e profissional dos participantes e um roteiro com seis questões disparadoras, elaboradas pelas autoras, relacionadas ao objeto de estudo, divididas em dois grandes domínios: a experiência no atendimento à PCR na unidade de APS; e a percepção crítica dessa experiência com ênfase nos aspectos relacionados ao talento humano e habilidades interpessoais.

A coleta de dados foi encerrada no momento em que todos os profissionais disponíveis na unidade, no período definido para coleta de dados, foram entrevistados. Não foi utilizada a técnica de saturação teórica dos dados para encerrar a pesquisa, mesmo com algumas respostas apresentando um padrão repetitivo, uma vez que o estudo pretendeu valorizar as experiências individuais e únicas dos participantes.

Os dados foram coletados individualmente após a assinatura do Termo de Consentimento Livre e Esclarecido em local escolhido pelo participante. As entrevistas foram audiogravadas, cuja duração aproximada foi de dez minutos e, posteriormente, transcritas para o banco de dados pessoal da equipe de pesquisa.

### Análise de dados

Utilizou-se o referencial metodológico de Bardin com a sistematização proposta por Oliveira^(17)^: pré-análise; exploração do material; e tratamento dos resultados. Após a transcrição das entrevistas, foi realizada a leitura flutuante do *corpus* de análise para iniciar a seleção das unidades de registro (URs), identificadas por meio de frases e unidades temáticas. A etapa de exploração do material envolveu a codificação de 523 URs, agregando a construção de 21 unidades de significação (USs) e o registro dessas unidades em tabelas (material suplementar). Em seguida, realizou-se o agrupamento das USs em três categorias temáticas.

## RESULTADOS

A amostra foi composta por 26 enfermeiros e nove técnicos de enfermagem. A Tabela 1 apresenta a caracterização dos participantes.

**Tabela 1:** Caracterização das participantes em duas unidades de Atenção Primária à Saúde, Brasil, 2024 (N=35)

Variável	Total n (%)
Feminino	32 (91,4%)
**Faixa etária** 18 a 30 anos 31 a 40 anos 41 a 50 anos 51 a 60 anos Maior de 60 anos	9 (25,7%) 13 (37,1%) 7 (20%) 5 (14,3%) 1 (2,9%)
**Categoria profissional** Enfermeira Técnica de enfermagem	26 (74,3%) 9 (25,7%)
**Tempo de formação** Menos de 1 ano 1 a 5 anos 6 a 10 anos 11 a 15 anos 16 a 20 anos Mais de 20 anos	1 (2,9%) 15 (42,9%) 5 (14,3%) 6 (17,1%) 5 (14,3%) 3 (8,6%)
**Tempo de atuação na APS** Menos de 1 ano 1 a 5 anos 6 a 10 anos 11 a 15 anos 16 a 20 anos Mais de 20 anos	7 (20%) 15 (37,1%) 6 (17,1%) 3 (14,3%) 2 (5,7%) 2 (5,7%)
**Atuou previamente na assistência hospitalar**	20 (57,1%)
**Presenciou uma PCR**	26 (74,3%)
**Participou de uma RCP**	19 (54,3%)
**Realizou treinamento prévio em SBV**	33 (94,3%)
**Realizou treinamento prévio em SAV**	8 (22,9%)

*APS - Atenção Primária à Saúde; PCR - parada cardiorrespiratória; RCP - reanimação cardiopulmonar; SBV - suporte básico de vida; SAV - suporte avançado de vida.*

A partir da análise de conteúdo temático categorial, foram obtidas quatro categorias temáticas, que serão apresentadas a seguir:

### Conhecimento e experiência como fatores determinantes no atendimento à pessoa em situação de parada cardiorrespiratória

Esta categoria é composta por 182 URs (34,8% das URs totais do *corpus* de análise) e dividida em duas subcategorias: “Conhecimento técnico e experiência prévia como fatores determinantes do sucesso da reanimação cardiopulmonar”; e “Educação permanente como estratégia de qualificação profissional”. Abarca quatro USs: “o conhecimento técnico como potencializador da assistência à PCR” (64 URs); “experiência prévia em RCP e vivência hospitalar como fatores determinantes para o atendimento” (14 URs); “não ocorrência de PCR e outras emergências de forma rotineira na APS” (33 URs); e “necessidade de treinamento periódico da equipe profissional da APS” (71 URs).

As URs a seguir evidenciam as falas das participantes quanto à categoria temática descrita:

*Se você não tem conhecimento daquela ação, do evento, você não consegue ter nenhum comportamento diferencial.* (E2)

*Primeiramente conhecer, ter domínio do conhecimento, e não só o profissional, mas todas as equipes que tiverem envolvidas, dominar o assunto. Acho que é fundamental para o atendimento ser de excelência.* (E8)

Os profissionais evidenciaram que possuir experiência prévia hospitalar ou em RCP é um fator determinante para o sucesso do atendimento à PCR na APS, ainda que o período da experiência tenha sido reportado como entre um e cinco anos para a maioria das participantes (57,9%).

*É uma característica daqui. A gente tem poucos profissionais que não têm experiência de hospital, que não trabalham em hospital. Mas o profissional que saiu da faculdade foi para atenção básica, nunca teve vivência de hospital, nunca viveu isso, assim, não vai rolar, vai ficar completamente disfuncional ali o trabalho, vai ficar completamente perdido.* (E10)

Sobre os desafios para realizar este atendimento na APS, os participantes apresentaram o treinamento periódico como fator determinante:

*Falta de treinamento periódico, então no município do* [omitido para preservação ética], *tem muita rotatividade na APS, então seria interessante esse treinamento acontecer sempre, não com tempo tão espaçado como acontece. Eu acho que isso poderia melhorar e não focar apenas em profissionais da saúde, e sim nos profissionais da unidade, desde o controlador de acesso até pessoas que trabalham na limpeza, administrativo também.* (E23)

*Treinamento diário, o que a gente muitas vezes não consegue fazer porque é muita demanda.* (E1)

Apesar de a maioria dos participantes afirmar já ter participado de alguma RCP (54,3%) e ter realizado treinamento prévio em suporte básico de vida (94,3%), suas falas evidenciam a insegurança em prestar este tipo de atendimento no contexto da APS. Um dos fatores que as participantes atribuem a esse resultado se relaciona à percepção de que esta não é uma situação rotineira nesse ponto de atenção.

*Principalmente por ser uma unidade de atenção básica. Assim, a gente não tem, digamos, o convívio com esse tipo de situação.* (E5)

*Porque não é algo que ocorre rotineiramente. Então, como não é que ocorre com frequência na nossa prática, às vezes, se ocorrer de repente, pode ser que haja alguma falha por conta disso.* (E11)

*Como não vivenciamos isso com frequência, às vezes pode ser um tanto complicado para ter aquele profissional bem qualificado para saber exatamente o que fazer.* (E33)

*Ter treinamento contínuo, porque, como não é uma rotina aqui, não é uma coisa que acontece o tempo todo, então os profissionais acabam não tendo a agilidade pra situação.* (E9)

### Macro e microprocessos de trabalho na Atenção Primária à Saúde: da chegada do usuário ao suporte avançado de vida

Esta categoria apresenta o processo de trabalho como fator determinante no atendimento à pessoa em situação de PCR. Composta por 159 URs (30,4% das URs totais), foi construída a partir de subcategorias intituladas: “Macroprocessos: rotina do serviço, demanda de trabalho e recursos materiais e estruturais”; e “Microprocessos: organização do ambiente de trabalho para o atendimento à pessoa em parada cardiorrespiratória”. Sua composição abarca seis USs, descritas a seguir.

A US “disponibilidade de material e estrutura física adequada na UBS” (84 URs) abarca as falas dos participantes quanto à necessidade de materiais e à infraestrutura, para que o atendimento seja realizado de forma rápida e eficiente:

*Primeira coisa, organização dos materiais e medicamentos, e que toda a equipe esteja ciente onde ficam os materiais.* (E11)

*Saber onde ficam os materiais, estrutura também da unidade, ter materiais disponíveis, fácil localização, fácil acesso, visualização, essas coisas.* (E14)

As USs “rotina de serviço e processo de trabalho na APS” (13 URs) e “demanda de trabalho na rotina da APS” (sete URs) destacam a rotina do serviço como um fator determinante para o atendimento, além de evidenciar que a demanda de trabalho também interfere nesse processo:

*Aqui, por exemplo, tem uma rotatividade muito grande de profissionais, aí a gente não sabe o preparo que eles têm.* (E18)

*A gente está no consultório fazendo pré-natal, fazendo puericultura, fazendo preventivo, aí manda um paciente passando mal, e aí tem que descer pra correr identificar que é uma parada ou não. Difícil pro pessoal que acolhe lá embaixo assim.* (E22)

A US “organização do setor e do fluxo de atendimento à PCR” (32 URs) aborda o microprocesso de trabalho inerente ao atendimento em si, cujos fatores determinantes podem agilizar ou atrasar o atendimento:

*Se você não parar e pensar, organizar o pensamento pra conseguir fazer todas essas etapas, você não vai sair dali, você vai travar, e aí você atrapalha todo o fluxo. Exatamente, todas as outras etapas.* (E13)

*Então, assim, a parada, por exemplo, tem que ter uma dinâmica onde todo mundo sabe o seu papel.* (E5)

A US “desconhecimento e desorganização do material e seu armazenamento como desafio no atendimento” (13 URs) se refere diretamente aos materiais e equipamentos necessários:

*Conferir se o material está todo completo, separado anteriormente. O profissional responsável tem que tá sempre fazendo checklist pra poder não ter nenhuma intercorrência durante o atendimento.* (E8)

A US “identificação precoce do quadro de risco” (dez URs) demonstra que a organização do microprocesso de trabalho tem início na identificação precoce da PCR:

*Eu acho que o que me dá mais medo na hora da atenção básica é a demora que pode acontecer esse reconhecimento do paciente chegar lá na frente passando mal e vir até* […] [a frase não é completada pelo participante]. *A gente fica muito dentro do consultório, né?* (E10)

### Habilidades comportamentais: *soft skills*

Esta categoria é composta por 182 URs (34,8% das URs totais) e dividida em duas subcategorias. A subcategoria 1 “Interfaces entre a liderança e a comunicação no atendimento à parada cardiorrespiratória” é composta por seis USs: “acolhimento e escuta qualificada” (três URs); “trabalho em equipe e boa relação interpessoal” (23 URs); “importância da comunicação efetiva entre a equipe para o sucesso da RCP” (dez URs); “atribuição da liderança da RCP a uma categoria profissional, principalmente enfermeiro e médico ou a quem iniciar o atendimento” (28 URs); “experiência e preparo como fatores essenciais para liderança” (18 URs); e “liderança como habilidade essencial durante o atendimento” (19 URs).

As URs a seguir evidenciam as falas dos participantes quanto à subcategoria descrita:

*Às vezes você está numa unidade básica onde você percebe às vezes uma dificuldade de relacionamento entre equipes, entre profissionais e eu acho que nesse momento, independente de adversidades, isso não pode acontecer. É esquecer qualquer adversidade e focar no que está acontecendo ali.* (E8)

*E eu acho que principalmente comunicação entre a equipe multiprofissional para atuar nesse tipo de cenário.* (E17)

*Assim, eu acho que o enfermeiro tem que ser* [o líder]*, porque o enfermeiro já está acostumado com isso, já está habituado com o gerenciamento de tudo. Então, a gente já tem essa prática, já é nossa, porque o enfermeiro conhece sala de procedimento, o enfermeiro sabe onde fica todos os materiais, o enfermeiro conhece todos os técnicos que estão aqui, sabe o técnico que é bom para pegar uma veia, sabe o técnico que é bom de fazer qualquer coisa, sabe o nome de todo mundo, “fulaninho de tal faz isso, fulaninho de tal faz isso”. Nem todo mundo, nem todas as categorias vão saber fazer isso.* (E10)

*O líder? A competência profissional, ele se torna líder. Ele está preparado, conforme eu falei. Ele tem que ter todo um conhecimento daquilo que tá fazendo para orientar a gente que é técnico a estar fazendo da forma correta.* (E31)

A subcategoria 2 “*Soft skills* em reanimação cardiopulmonar na Atenção Primária à Saúde” é composta por cinco USs: “a importância da agilidade no sucesso da RCP” (22 URs); “ter segurança para realizar o atendimento” (dez URs); “ter controle emocional durante o atendimento” (28 URs); “o não pertencimento dos profissionais de APS na RUE e nos elos da cadeia de sobrevivência” (17 URs); e “necessidade de foco e atenção no atendimento à PCR” (quatro URs).

As seguintes asserções corroboram a subcategoria descrita:

*Eu acho que proatividade. Agilidade para você pensar rápido, para você agir rápido. Atenção, foco. Acho que é isso.* (E22)

*Calma. Calma e saber raciocinar, porque, na hora do desespero, as pessoas batem cabeça e acaba não dando certo. Infelizmente, num momento super caótico, você vai ter que ser a pessoa calma; tem que ter duas pessoas calmas. Calma no sentido, assim, de saber direcionar. É lógico que a tensão está, mas assim, de dar o comando saber como lidar com aquilo. Então, isso acho que não é nem calma, digamos, é saber direcionar realmente. A segurança tem que passar segurança para todos que estão ali.* (E20)

*Porque normalmente, assim, a gente orienta a população, mas a população não sabe que aqui não é um lugar de urgência e emergência. Então, se parar vem pra cá. É difícil*. (E5)

O Quadro 1 (material complementar) sintetiza a elaboração das quatro categorias temáticas, incluindo as URs e USs, obtidas a partir da análise do conteúdo.

### DISCUSSÃO

O presente estudo apresenta os fatores determinantes e as *soft skills* no atendimento à pessoa em situação de PCR na APS. Trata-se de contexto pouco explorado para esse tipo de tema e prática, porém com grande potencial de reverter o quadro de PCR, uma vez que está localizado próximo à residência e organizado de forma comunitária.

A insegurança dos profissionais pode ser justificada pois sabe-se que, quanto menos frequente o contato, menor é a retenção do conhecimento e das habilidades^(18)^. Treinamentos esporádicos podem não ser o suficiente para a efetiva atuação no atendimento à PCR^(19,20)^. Dessa forma, recomendam-se ações de educação permanente teórico-prática com maior frequência e menor intensidade de novos conhecimentos - “*low dose - high frequency model*”^(21)^.

Além da abordagem teórica e a prática da técnica de RCP, nossos resultados indicam que as estratégias de educação permanente dos profissionais de saúde precisam incluir planos que possibilitem a aquisição de habilidades comportamentais aplicadas ao local de trabalho e recursos disponíveis, sem impor pressão excessiva. São possibilidades de inovação a inclusão de pacientes e famílias para o empoderamento dos profissionais^(22)^, a integração entre encontros presenciais e uso de tecnologias como vídeos e aulas remotas^(23)^, o uso de jogos interativos^(24)^, e *smart devices*, como celulares/aplicativos ou relógios^(25)^.

A categoria “Macro e microprocessos de trabalho na Atenção Primária à Saúde: da chegada do usuário ao suporte avançado de vida” traz como principais fatores determinantes a disponibilidade de materiais, a infraestrutura adequada, a rotina de serviço e o processo de trabalho na APS. Destacam-se aqui a demanda de trabalho na rotina da unidade, a organização do setor e do fluxo de atendimento à PCR, a identificação precoce do quadro de risco, e o desconhecimento sobre o material e seu armazenamento como desafios no atendimento.

É consenso que a disponibilidade de materiais e equipamentos é um fator indispensável ao atendimento à vítima de PCR. Por isso, é essencial assegurar sua provisão necessária e organização. No entanto, a insuficiência de materiais ou insumos não deve prorrogar ou interromper a reanimação^(20)^. As ações realizadas durante os minutos iniciais de atendimento à PCR são críticas em relação à sobrevivência; logo, o suporte básico de vida deve ser realizado de maneira adequada e rápida^(1)^. Há, portanto, uma corresponsabilização entre a ação institucional e as *soft skills* individuais e interpessoais.

A última categoria agrupou as *soft skills,* que são as habilidades comportamentais - individuais e sociais - relacionadas à maneira como o profissional lida com o outro e consigo em diferentes situações. São frequentemente agrupadas em três categorias: competências sociais, como a liderança; competências cognitivas, como a consciência situacional; e fatores de recursos pessoais, como a tomada de decisão diante de situações de estresse^(26)^.

No contexto do atendimento à PCR na APS, essas habilidades podem determinar a eficiência do atendimento, o que modifica desfechos clínicos e satisfação profissional^(27)^. Emergiram, neste estudo, *soft skills* relacionadas à liderança, agilidade, segurança, controle emocional, foco, e atenção e sensação de pertencimento.

Os participantes apontaram a liderança como um fator essencial para o êxito do atendimento. O profissional que detém conhecimento técnico possui mais segurança para liderar o processo de forma qualificada. O êxito da equipe no atendimento de emergência depende da eficiência de um líder para orientar os profissionais envolvidos no atendimento^(28)^.

Há relação direta entre a capacidade de comunicação do líder e o desempenho da equipe, onde a comunicação efetiva, a habilidade técnica e um bom relacionamento interpessoal compõem a tríade necessária à liderança efetiva condução da RCP^(28)^. Assim como evidenciado nas entrevistas, a literatura também aponta o enfermeiro como elo fundamental na integração e organização da equipe, facilitando os processos de trabalho, além de participar das atividades desenvolvidas e fornecer suporte teórico aos membros através da educação permanente^(29)^.

A agilidade é exigida nos protocolos clínicos de atendimento à PCR, tanto para o rápido reconhecimento da situação quanto para início da RCP e desfibrilação precoce quando indicada. A agilidade relaciona-se intimamente à segurança em realizar uma ação e o foco ao que se implementa. Essas habilidades tornam-se ainda mais importantes no contexto da APS, visto que nem sempre a unidade de saúde está preparada para o atendimento de emergências.

Avançar na formação com vistas a promover a aquisição de *soft skills* é um desafio contemporâneo que vem sendo alvo de estudos recentes^(30-32)^. Nesse sentido, a constante atualização profissional e a análise da incorporação de diferentes métodos para o desenvolvimento de habilidades não técnicas no atendimento à PCR são campos em potencial para pesquisas futuras.

Além disso, incluir atividades que possibilitem o desenvolvimento das *soft skills* no contexto do atendimento à pessoa em situação de PCR na APS merece atenção nos espaços de ensino e nas políticas públicas de saúde. Ainda, o próprio facilitador de treinamentos voltados à educação permanente para RCP precisa se apoiar também em *soft skills* ^(26)^.

### Limitações do estudo

O estudo apresenta como limitações a inclusão de apenas duas unidades de ESF, que se localizam relativamente próximas às unidades de pronto atendimento, o que, supostamente, leva à redução da vivência do atendimento à PCR pelos participantes. Ainda, uma limitação metodológica refere-se à ausência de confirmação dos dados coletados após a transcrição com as participantes.

### Contribuições para as áreas da enfermagem, saúde ou políticas públicas

A presente pesquisa evidencia a fragilidade do desenvolvimento de *soft skills* entre profissionais de enfermagem da APS para o atendimento à pessoa em situação de PCR. Nesse sentido, é um desafio atual aprimorar as políticas públicas e os centros de formação com metodologias adequadas e inovadoras que possibilitem a aquisição de habilidades não técnicas para o suporte básico e avançado de vida. As metodologias ativas de ensino, sobretudo a simulação realística em saúde, podem trazer grandes contribuições para a superação desse desafio.

Os resultados desta pesquisa poderão apoiar o desenvolvimento de intervenções voltadas ao aprimoramento do conhecimento teórico-prático e de habilidades comportamentais dos profissionais da APS, direcionando as ações de educação permanente das equipes e implementação de ações por *stakeholders* vinculados à Rede de Atenção à Saúde. Ainda, este estudo aponta as habilidades necessárias para o atendimento à pessoa em PCR no contexto da APS, a partir da identificação pela própria enfermagem, de modo a guiar estudos futuros que avaliem as diferentes metodologias e os desfechos relacionados à RCP.

## CONSIDERAÇÕES FINAIS

Este estudo identificou categorias temáticas relacionadas aos fatores determinantes e às *soft skills* na percepção da equipe de enfermagem atuante na APS diante da situação de PCR. Destaca-se a importância da liderança, comunicação efetiva, tomada de decisão rápida, ágil e focada, e a organização do processo de trabalho como habilidades comportamentais não técnicas fundamentais para o sucesso da RCP nesse contexto. A corresponsabilidade para o sucesso no atendimento à pessoa em situação de PCR na APS também é evidenciada pela necessidade da disponibilidade de materiais, infraestrutura adequada, organização da rotina de serviço e processo de trabalho. Estudos que avaliem as *soft skills* (habilidades comportamentais e interpessoais) em contextos sociais diferentes são encorajados.

## Data Availability

Os dados de pesquisa só estão disponíveis mediante solicitação.
